# Perspectives on Pertussis Prevention

**DOI:** 10.1111/apa.70377

**Published:** 2025-11-19

**Authors:** Ulrich Heininger

**Affiliations:** ^1^ University Children's Hospital Basel Basel Switzerland; ^2^ Faculty of Medicine University of Basel Basel Switzerland

Pertussis occurs worldwide in patients of all ages but is most serious in young infants who can die from apnea and respiratory failure induced by hyperleukocytosis [1]. Treatment of pertussis is difficult as it is usually initiated too late during the course of the disease to prevent complications. Also, prevention of pertussis in young infants by avoidance of exposure, post‐exposure antibiotic prophylaxis, or indirect protection by immunisation of close contact persons (so‐called “cocooning”) has been shown to be impossible or at least extremely challenging. Therefore, direct protection by immunisation remains the main cornerstone of prevention of pertussis.

Immunisation against pertussis has come a long way since the invention of the first vaccines approximately 100 years ago (summarised in [1]). The first generation was whole‐cell vaccines (WCV), consisting of killed *Bordetella pertussis (Bp)* bacteria. Due to their high reactogenicity and alleged (but later on unjustified) claims that they would cause brain damage in infants, second generation vaccines, named acellular (AV), consisting of pertussis toxoid (PT) and up to four other *Bp* proteins, were developed in the 1970s in Japan and licensed in many countries worldwide after successful efficacy trials had been performed in the 1990s. Of note, neither WCV nor AV provide long‐term effectiveness, i.e., booster doses are required to maintain protection, and initial protection is suboptimal with effectiveness estimates in the range of approximately 80%–95% against typical pertussis (defined as prolonged cough over at least 2 or 3 weeks with paroxysm, whoop, and/or vomiting) and substantially lower against less typical disease manifestations [1]. A major advancement over the last approximately 15 years in protecting young infants against severe disease has been the introduction of pertussis vaccination in pregnancy (VIP), thereby preventing life‐threatening hyperleukocytosis associated with *Bp* infection before having received their own primary vaccine series [2].

Where do we go from here, and what should be done to better control pertussis in the future?

There are several areas of potential improvement in the current situation, which can be reached with some common efforts, as follows.

## 1 Pertussis Vaccination Should Be Offered for Free in Every Pregnancy Worldwide

Transplacental passive immunisation after immunisation in the second or third trimester of every pregnancy provides the newborn with sufficient amounts of protective anti‐PT IgG serum antibodies in utero. Its clinical effectiveness has been demonstrated in the United Kingdom, where a first pertussis VIP program was introduced in October 2012 [2], and elsewhere. Infants, whose mothers were immunised during pregnancy at least 14 days before delivery, had at least a 90% lower risk of acquiring pertussis in the first few months of life in comparison with infants born to unimmunised mothers.

However, the optimal timing of VIP is still a matter of debate. In this regard, vaccination late in the second or early during the third trimester appears to be ideal to protect infants, including the majority of infants born preterm. VIP is a measure that is safe for the infant and the mother, even if she receives it repeatedly during sequential pregnancies within a few years [3]. It has been shown with different AV formulations that anti‐*Bp* IgG antibodies against all *Bp* antigens included in the respective vaccine increased 1 month after vaccination in the pregnant woman but were then followed by a rapid decline within the first year after administration. Therefore, administering AV only during the first pregnancy would not guarantee high enough amounts of specific IgG antibodies, especially against PT, for the foetus in a subsequent pregnancy. Unfortunately, in most countries worldwide today, AV is only available in combination with tetanus (T) and diphtheria (d) toxoid vaccines, collectively named “Tdap”. It should be noted that repeated T and d booster doses are not necessary for the protection of the mother (and her infant). Therefore, stand‐alone pertussis vaccines would be sufficient to fulfil the primary purpose of protecting the infant from pertussis. Although no safety issues have been noted so far when Tdap was administered in repetitive pregnancies within a few years, I am convinced that the broad availability of stand‐alone vaccines without T and d toxoids would improve acceptance of pertussis vaccination in every pregnancy. As of August 2025, only one such product, manufactured and distributed in Thailand, is currently available. Given the high burden of pertussis disease, specifically in infants, pertussis vaccination in every pregnancy, either as Tdap or AV alone, should become the gold standard and be made possible for free all over the world, which has been identified as a major goal in the field of pertussis control by the Global Pertussis Initiative (GPI) [4].

## 2 Timely and Complete Immunisation in Infants Is Key

In addition to VIP, pertussis primary immunisation (in combination with other vaccine antigen components such as *Haemophilus influenzae* type b, inactivated poliomyelitis viruses and hepatitis B surface antigen, as recommended nationally) should be initiated and completed as soon as possible. This ideally requires 2 or 3 doses with 2‐ and 1‐month intervals, respectively, and the first dose given at 6–8 weeks of life. A further dose (called “+1”) at least 6 months later, i.e., at 10–12 months of age, will complete the primary “2 + 1” or “3 + 1” immunisation series. Although challenging from a programmatic point of view, 2 + 1 or 3 + 1 immunisation schedules could also be preferred in low‐ and middle‐income countries (LMIC) compared to the currently recommended 3 + 0 schedule at 6, 10 and 14 weeks of age by WHO as part of their expanded program on immunisation (EPI). Currently, most LMIC rely on WCV for their infant pertussis immunisation programs, as recommended by WHO. In this context, it is often postulated that the duration of protection would last longer after WCV than after AV. However, there is no evidence for this. In contrast, waning immunity has also been shown for WCV [5]. Today, many LMIC have mixed markets, i.e., AV products are also licensed and available on the private market [6]. In my view, whenever possible, AV should be preferred over WCV not only for booster doses but also for primary immunisation due to their better tolerability and standardised composition.

Before the COVID‐19 pandemic, the coverage with 3 doses of the Diphtheria–Tetanus–Pertussis vaccine had gradually increased worldwide but experienced a severe setback during the pandemic, mainly due to the interference of measures to fight the spread of SARS‐CoV‐2 with access to vaccination clinics in many parts of the world [7]. It has been challenging ever since (and unfortunately will remain so for the foreseeable future) to return to previous coverage rates, let alone to further improve them.

## 3 Lifelong Pertussis Vaccination Is Advantageous, and Better Pertussis Vaccines Are Needed

In contrast to many other vaccines—such as those against tetanus, measles and hepatitis, for example—AV and WCV in current use are of suboptimal effectiveness. This means primary (despite complete immunisation) and secondary vaccine failures (due to waning immunity with increasing time after primary immunisation) are common in many places with high coverage rates and/or lack of regular booster doses throughout childhood, adolescence, and adulthood. Taken together, this is a frequent cause of confusion about their usefulness in general. Therefore, two important measures are desirable, independent of each other:

First, to reduce the circulation of *Bp* in the population and thereby decrease exposure of individuals with primary or secondary lack of protection to pertussis, regular booster doses with AV are advantageous. Yet only a few countries have introduced booster doses in pre‐school and/or adolescent age groups, let alone in adults, so far. Based on observational epidemiologic studies, a booster dose should ideally be recommended every 5 years throughout life if optimal control of pertussis with currently available AV is the goal. This booster interval is clearly shorter than those which are required to maintain protection against tetanus and diphtheria, which again explains the desire for stand‐alone AV in addition to Tdap combination vaccines.

Second, better pertussis vaccines are needed. It is unclear why the currently used WCV and AV do not provide optimal protection and how they could be improved. One approach followed by a consortium named PERISCOPE is to try to identify biological markers that would allow establishing a correlate of protection, which could guide vaccine manufacturers in improving the antigen composition of AV in the future [8]. This work is still in progress. A second promising improvement could be a *Bp* live, attenuated vaccine. One such vaccine, termed BPZE1, is administered intranasally and induces both mucosal and systemic immune responses [7]. It has theoretical potential for more effective and sustained immunity compared to current WCV and AV, which are inactivated or purified pertussis antigens, respectively. After administration, BPZE1 colonises the respiratory tract of the mice and protects them against challenge with virulent *Bp*. Preliminary immunogenicity and reactogenicity studies have been performed in school‐age children and adults (see https://clinicaltrials.gov) with promising results [9], but none have evaluated its clinical effectiveness.

In conclusion, the future of pertussis control by immunisation offers promising aspects. On the one hand, the use of currently available acellular and, if these are not affordable in a given country, whole‐cell pertussis vaccines in infancy should be optimised. This means, timely start and completion of the immunisation series—with the last of 3 or 4 doses administered with an expanded interval after the previous dose for optimal immunogenicity—should be attempted. Moreover, coverage should be as close to 100% as possible for all infants worldwide. On the other hand, early protection after birth is pivotal, and this can only be achieved by passive immunisation via the placenta, i.e., VIP. Investment in an acellular vaccine for every pregnancy and everywhere is promising to be cost‐efficient and lifesaving. Finally, one or more pertussis vaccines with improved effectiveness compared to currently used AV and WCV would be a game changer and could reduce the remaining burden of pertussis disease in infants caused by primary vaccine failure. Common efforts in each of these areas are needed and will be highly welcome.

## Conflicts of Interest

Ulrich Heininger is a member of the Global Pertussis Initiative (GPI) and has received honoraria for participation in annual GPI meetings by Sanofi (France).

## Data Availability

Data sharing not applicable to this article as no datasets were generated or analysed during the current study.
